# Multi-Dose Intravenous Administration of Neutral and Cationic Liposomes in Mice: An Extensive Toxicity Study

**DOI:** 10.3390/ph15060761

**Published:** 2022-06-18

**Authors:** Stéphanie Andrade, Joana A. Loureiro, Santiago Ramirez, Celso S. G. Catumbela, Claudio Soto, Rodrigo Morales, Maria Carmo Pereira

**Affiliations:** 1 LEPABE, Department of Chemical Engineering, Faculty of Engineering, University of Porto, 4200-465 Porto, Portugal; stephanie@fe.up.pt (S.A.); jasl@fe.up.pt (J.A.L.); 2 ALiCE—Associate Laboratory in Chemical Engineering, Faculty of Engineering, University of Porto, Rua Dr. Roberto Frias, 4200-465 Porto, Portugal; 3 Department of Neurology, The University of Texas Health Science Center at Houston, 6431 Fannin St., Houston, TX 77030, USA; santiago.d.ramirez@uth.tmc.edu (S.R.); celso.catumbela@uth.tmc.edu (C.S.G.C.); claudio.soto@uth.tmc.edu (C.S.); 4 Centro Integrativo de Biologia y Quimica Aplicada (CIBQA), Universidad Bernardo O’Higgins, Santiago 1497, Chile

**Keywords:** lipid-based nanoparticles, nanocarrier, surface charge, delivery systems, chronic treatment, mice

## Abstract

Liposomes are widely used as delivery systems for therapeutic purposes. However, the toxicity associated with the multi-dose administration of these nanoparticles is not fully elucidated. Here, we evaluated the toxicity of the prolonged administration of liposomes composed of neutral or cationic phospholipids often used in drug and gene delivery. For that purpose, adult wild-type mice (C57Bl6) were randomly distributed into three groups receiving either vehicle (PBS), neutral, or cationic liposomes and subjected to repeated intravenous injections for a total of 10 doses administered over 3 weeks. Several parameters, including mortality, body weight, and glucose levels, were monitored throughout the trial. While these variables did not change in the group treated with neutral liposomes, the group treated with the positively charged liposomes displayed a mortality rate of 45% after 10 doses of administration. Additional urinalysis, blood tests, and behavioral assays to evaluate impairments of motor functions or lesions in major organs were also performed. The cationic group showed less forelimb peak force than the control group, alterations at the hematological level, and inflammatory components, unlike the neutral group. Overall, the results demonstrate that cationic liposomes are toxic for multi-dose administration, while the neutral liposomes did not induce changes associated with toxicity. Therefore, our results support the use of the well-known neutral liposomes as safe drug shuttles, even when repetitive administrations are needed.

## 1. Introduction

The poor pharmacokinetics, reduced bioavailability, and high toxicity of most therapeutic molecules are some of the aspects that decrease their therapeutic efficacy. Such limitations can be overcome using delivery systems to protect molecules from degradation and direct them to the desired target site [1].

Liposomes are spherical vesicles composed of phospholipid bilayers enclosing aqueous compartments [2]. The amphiphilic property of phospholipids, which display hydrophilic polar heads and lipophilic tails, allows the encapsulation of hydrophilic compounds in the aqueous space and lipophilic compounds in the lipid bilayer [3]. The biocompatibility, bioavailability, high loading capacity, ease of production, and sustained release of therapeutic agents are other properties that stand out [4]. Conventional liposomes composed of neutral lipids were the first generation of lipid vesicles to be used by the pharmaceutical industry. In turn, cationic liposomes represent the newest generation of liposomes and have been used in gene therapy [5].

Despite several advantages related to the use of liposomes as delivery systems, they have some drawbacks that restrict their therapeutic potential. While liposomes have been successfully used to reduce the toxicity of therapeutic agents, the vesicles themselves can induce toxicity. Dozens of in vivo studies have described the toxicity associated with the administration of one or just a few doses of cationic liposomes [6,7,8]. However, these reports lack information regarding the long-term toxicity, which is crucial considering the therapeutic regime of several pathologies, such as chronic diseases, that require the repeated administration of therapeutic agents to ensure sustained drug levels for extended periods [9].

Thereby, we conducted the first prolonged systemic toxicity study by intravenously administering 10 doses of neutral and cationic liposomes to wild-type mice over 3 weeks to better understand the effect of multi-doses administration of this lipid based nanocarriers in vivo. Bare NPs were tested without any targeting ligand to focus this study on evaluating the nanocarriers’ toxicity upon repeated administration. Lipid vesicles were characterized physicochemically in terms of hydrodynamic diameter, polydispersity index (PDI), and zeta potential. The long-term stability at storage conditions was also evaluated for over 4 months. Mice mortality, variations in body weights, glucose levels, motor abilities, microscopic urinalyses, liposome–blood interaction, and organ weight and morphology were assessed to evaluate potential liposome-induced toxic effects. Histopathological examinations were also performed, and liver injury markers in blood were quantified.

## 2. Results and Discussion

### 2.1. Physicochemical Characterization of Liposomes

The physicochemical properties of the neutral and cationic LUVs were evaluated in terms of hydrodynamic diameter, PDI, and zeta potential values, as shown in Table 1. While neutral liposomes exhibited a hydrodynamic diameter of 139 ± 13 nm, the cationic liposomes showed a significantly smaller average of 112 ± 3 nm (*p* < 0.05) (Table 1). Such a difference may be related to the distinct composition of the liposomes [10].

It has been reported that the efficacy of a delivery system is closely related to the size of NPs since this property can affect the in vivo stability, blood circulation time, agent release, cell uptake, clearance, and toxicity of NPs. NPs larger than 200 nm have revealed to be quickly cleared from the bloodstream by the lymphatic system [11]. However, nanocarriers too small have been connected to higher toxicity as the NP surface area increases. Thus, an optimal diameter of around 100 nm has been identified, as particles around that size have shown decreased toxicity [1]. Such evidence proved that the produced formulations have mean sizes valid for delivery applications.

The size distribution of NPs was evaluated by assessing the PDI of formulations. All formulations presented PDI values lower than 0.2 (Table 1), suggesting that the lipid suspensions have homogenous sizes, and therefore are valid for delivery purposes [12]. However, the neutral liposomes showed PDI values significantly higher than the cationic liposomes (*p* < 0.05), probably due to the different techniques used to reduce the size of the vesicles. This data goes in line with a report by Ong et al., demonstrating that extruded liposomes present smaller PDI values than sonicated liposomes [13].

The surface charge of NPs is another key property to consider when designing delivery systems since it affects nanocarriers’ in vivo internalization rate and toxicity. As expected, neutral LUVs showed a zeta potential close to zero mV, while cationic LUVs exhibited a zeta potential of 33 ± 2 mV (Table 1). 

**Figure 1:**
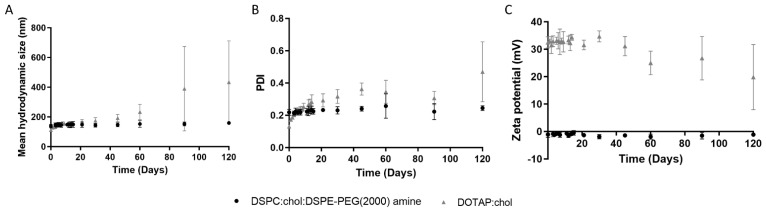
Physicochemical characterization of liposomes over 4 months in terms of (**A**) hydrodynamic diameter, (**B**) PDI, and (**C**) zeta potential values.

Liposome stability remains a major limitation for their clinical application. For that reason, it is crucial to ensure their stability during the production process and the duration of the experiments, since NPs tend to aggregate to attain a thermodynamically favorable state. If a NP product is expected to be commercialized, its physicochemical properties should be preserved during the storage period. Hence, a long-term stability study of the produced liposomes was performed at storage conditions (4 °C) over 4 months (Figure 1). Variations in some of these characteristics suggest that the structure of NPs is altered over time, which may be linked with the loss of their stability and potential activities [14]. From Figure 1, it is possible to observe that the physicochemical properties of the neutral liposomes remained constant (*p* > 0.05) for at least 4 months when stored at 4 °C in the buffered medium. Moreover, while neutral LUVs exhibited a mean d/d_0_ value of approximately 1.1 over the 4 months study period, cationic vesicles presented a value of 3.9 at the experimental endpoint, indicating NPs aggregation. The d/d_0_ value represents the ratio between the mean diameter of the NPs at each time point and the initial mean diameter, being an indicator of size variation [15]. However, the physicochemical properties of DOTAP:CHOL vesicles were not significantly changed over the first month of storage. After this time, cationic liposomes exhibited a mean diameter above 200 nm and a PDI over 0.4.

### 2.2. Mice Survival and Clinical Observations

**Figure 2:**
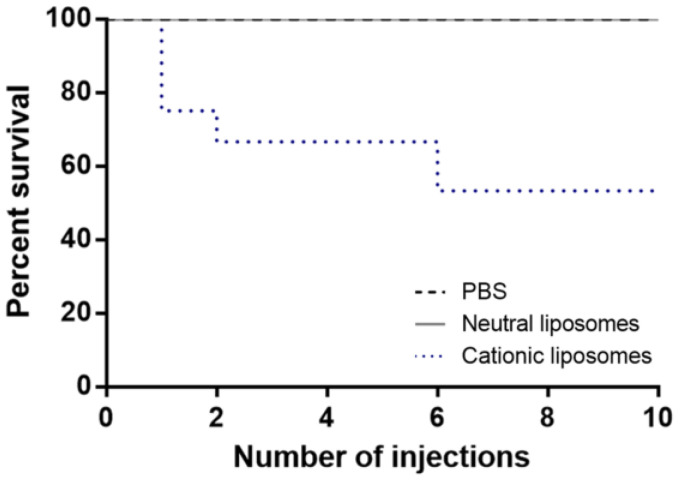
Effect of the repeated administration of PBS, neutral and cationic liposomes on mice survival.

The survival curves of mice repeatedly exposed to the different liposomes are shown in Figure 2. No mortality was registered among mice exposed to repeated doses of neutral liposomes, thus overlapping the survival curve of the control group (treated with PBS). However, a significant mortality rate was observed in mice treated with cationic liposomes, with an average of 27% mortality immediately after the first injection. Moreover, the mortality rate increased to 36% after the second injection. From the sixth to the tenth administration, mice treated with cationic liposomes displayed a survival rate of 55%. These results are in accordance with Chien et al. (2005) [16], which observed a 33% mortality rate after injecting three doses of cationic liposomes to male BALB/c mice. Despite the mortality observed in mice exposed to cationic liposomes, none exhibited alterations in fur appearance, eyes, sleep cycles, salivation, defecation, food and water intake, or other visible signs.

### 2.3. Bodyweight

Bodyweight is frequently recorded in toxicology studies, since variations over 20% can be linked with toxic effects [17]. Thus, mice body weight was recorded before each administration and euthanasia (Supplemental Table S1). At the baseline, the PBS, neutral liposomes, and cationic liposomes groups showed an average body weight of 21.6 ± 1.3, 21.6 ± 1.4, and 21.2 ± 0.8, respectively, with no significant differences between the liposomes-treated and control mice (*p* > 0.05). Mice body weight remained stable until the end of the experiment (*p* > 0.05). Similar findings were obtained by Knudsen et al. (2015) [18] after administering a single dose of cationic liposomes to male Han Wistar rats.

### 2.4. Glucose Levels

Blood glucose represents another extensively used indicator in toxicological studies since variations in glucose levels are an established toxicity surrogate. Importantly, fluctuations in blood glucose concentrations can induce secondary toxic events, including glial toxicity, oxidative stress, and inflammatory processes [19]. Thus, the glucose levels of mice treated with repeated doses of PBS, neutral-, and cationic-lipid vesicles were monitored weekly and are shown in Supplemental Figure S1. Before starting the injection of LUVs, control, neutral, and cationic groups exhibited glucose levels of 135 ± 14, 126 ± 8, and 140 ± 10 mg/dL, respectively, with no significant differences between groups (*p* > 0.05). No significant variations were observed over the experiment in neutral and cationic liposomes-treated mice compared with the control group (*p* > 0.05), which is in concordance with a previous study [18].

### 2.5. Behavioral Tests

**Figure 3:**
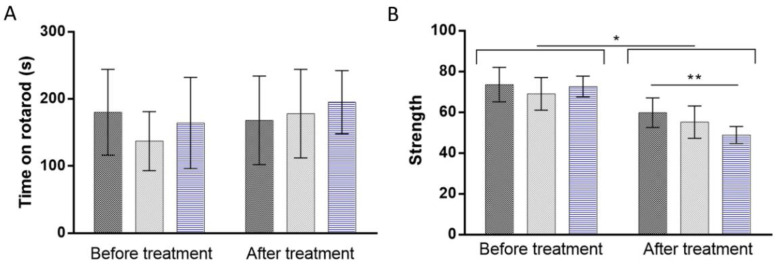
Effect of multi-dose administration of PBS (dark gray), neutral (light gray), and cationic liposomes (blue) on (**A**) rotarod and (**B**) grip strength performance. * *p* < 0.05, ** *p* < 0.01.

Behavioral monitoring is a sensitive way to assess the nervous system toxicosis induced by a tested drug or material [20]. In fact, variations in the behavioral responses of animals may be related to the impairment of sensory, motor, and cognitive aspects. The rotarod test is currently one of the most used behavior tests where the neurotoxicity or effect of a compound on animal behavior can be evaluated [21]. By monitoring the time rodents remain in the rotarod, motor coordination and balance defects can be detected [22]. Therefore, the rotarod test was performed before starting the NPs injection and 24 h-post-treatment. To complement the rotarod test, a forelimb grip strength test was performed 48 h post-treatment to evaluate the limb motor and neuromuscular function of experimental and control rodents [23]. The results of the rotarod and grip strength tests are shown in Figure 3A,B, respectively.

Concerning the rotarod test (Figure 3A), no significant variation in the latency to fall from the rotarod apparatus was detected after the repeated administration of both neutral and cationic vesicles compared to the control group (*p* > 0.05). These data suggest the absence of motor dysfunction due to liposome treatments. However, grip strength data (Figure 3B) revealed a significant reduction of the maximal muscle strength of mice after the repeated injection of PBS, neutral, and cationic liposomes (*p* < 0.05). Although variations on the maximal peak force may indicate motor neurotoxicity, the decreased grip strength of the control group suggests a lack of interest in the trial, potentially due to the excessive manipulation associated with repeated injections. Alternatively, rodents may get used to the manipulation, reducing their willingness to execute the task. Regardless, the repeated administration of cationic liposomes significantly declined the forelimb peak force values of mice by around 9.1% compared to the control group (*p* < 0.01), suggesting that these particular vesicles induce harmful effects on limb motor and neuromuscular function of mice.

### 2.6. Microscopic Urinalysis

The microscopic examination of urine is another common procedure to detect possible toxicity signals. Urinalysis identifies abnormal solutes, cells, casts, crystals, organisms, or particulate matter that may indicate some renal or systemic pathology. Supplemental Figure S2 shows the microscopic urinalysis of mice after the prolonged treatment of PBS, neutral, or positively charged liposomes. The urine of healthy animals or patients usually contains several chemicals that can be found in the form of crystals, and so, a small number of urine crystals become clinically irrelevant. However, the presence of abnormal crystals may indicate renal dysfunction. Some usually present crystals (triple phosphate) are pointed by black arrows in Supplemental Figure S2. In contrast, evidence of hippuric acid crystals was identified in the urine of mice treated with neutral liposomes.

In addition to crystals, some endothelial cells are also commonly present in the urine of healthy individuals. Red arrows in Supplemental Figure S2 depict some of these cells. However, other cell types are not expected to be found in urine, and thus, their presence is acknowledged as an indicator of health issues. An example of this are red blood cells (RBCs). When present in urine, RBCs are indicative of a disease condition known as hematuria. Supplemental Figure S2C (blue arrows) indicates some of the numerous RBCs present in the urine of mice treated with cationic LUVs, indicating damage at this level. Notably, hematuria was absent in the urine of the control and neutral liposome groups. Importantly, the RBCs identified in the cationic LUVs-treated mice’s urine exhibited an unusual form with a spiked cell membrane, characteristic of acanthocytes [24]. This abnormal kind of RBCs possesses some spikes of varying lengths and widths irregularly located on the cell surface, as shown by the green arrows in Supplemental Figure S2D.

### 2.7. Liposomes–Blood Interaction

**Figure 4:**
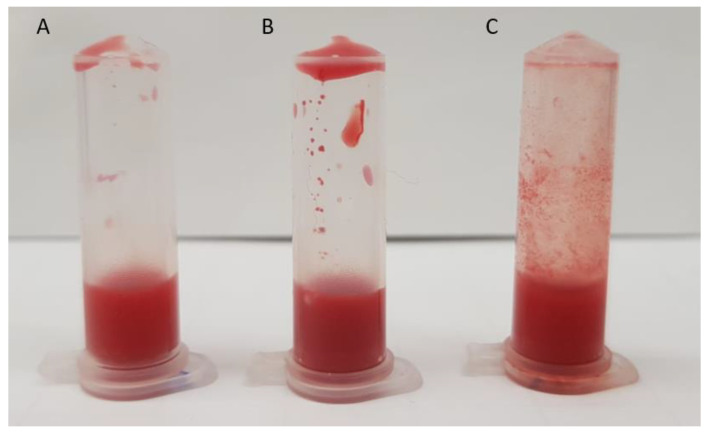
Ex vivo blood–liposome interaction. Blood from non-treated mice was mixed with either (**A**) PBS, (**B**) neutral, or (**C**) cationic liposomes.

Before reaching target tissues or cells, liposomes injected intravenously first interact with blood components [25]. Thus, after observing a 27% mortality rate caused by the first injection of DOTAP:CHOL LUVs, an in vitro LUVs-blood interaction study was performed to identify the possible cause of such immediate deaths. Figure 4 shows blood samples from untreated mice mixed with PBS, neutral, or cationic liposomes. It is possible to observe that positively charged liposomes induced noticeable changes in blood, substantially increasing its turbidity and inducing coagulation (Figure 4C). In contrast, neither the increase of the turbidity nor the formation of clots was verified when PBS and neutral liposomes were mixed with blood (Figure 4A,B). Confirming the previous observations, a microscopic analysis of blood smears suggests that RBCs agglutinate in the presence of cationic liposomes (Supplemental Figure S3). These findings are in line with Senior’s report [26], which also detected the increase in turbidity and the formation of clot-like mass upon the incubation of cationic liposomes with rat plasma. Furthermore, the authors revealed that the extent of plasma–liposomes interactions depend on the concentration of the phospholipid positive charge [26].

The acute interaction between blood components and liposomes was also assessed in vivo by intravenously injecting one dose of DOTAP:CHOL LUVs into mice. Therefore, fresh blood was collected, and a blood smear was performed on a glass slides to analyze RBCs morphological alterations, several regions of the slide were observed, and the diameter or RBCs was measured in pixels using image software. The microscopic analysis of the blood smears of these mice shows that, after a single administration, positively charged liposomes induce morphological changes in RBCs (Supplemental Figure S4). Specifically, RBCs transitioned from a regular spherical shape (Supplemental Figure S4A) to irregular structures involving either fusiform (acuminocytes) (Supplemental Figure S4B, red arrows) or teardrop forms (dacrocytes) (Supplemental Figure S4B, blue arrows).

**Figure 5:**
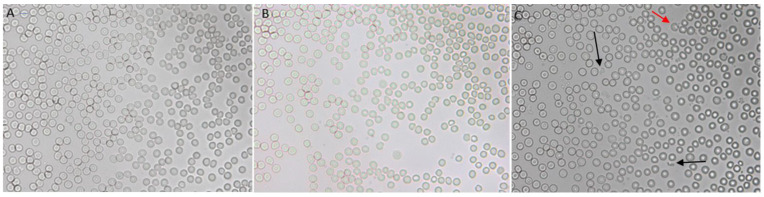
In vivo cationic liposomes–blood interaction. Microscopic images of blood smears from mice treated with (**A**) PBS, (**B**) neutral, and (**C**) cationic liposomes. Images obtained at a 40× magnification. Black and red identify schistocytes and hemolysis, respectively.

While numerous acuminocytes and dacrocytes were observed after a single dose of DOTAP:CHOL LUVs (Supplemental Figure S4B), such variations were less evident after repeated administration of cationic liposomes (Figure 5C). Instead, several fragmented RBCs (commonly called schistocytes) were identified in the blood smears of mice from the cationic liposomes group (Figure 5C, black arrows). Such structures are smaller than usual RBCs and typically irregularly shaped, and result from hemolysis (Figure 5C, red arrow). Hemolysis of erythrocytes caused by the interaction with cationic liposomes was previously observed in vitro [26]. No irregular structures were noticed in the groups treated with PBS (Figure 5A) and neutral liposomes (Figure 5B). Moreover, we observed high variability in the size of RBCs, a condition called anisocytosis (Supplemental Figure S5). Anisocytosis was quantified by assessing the RBC’s mean width and the standard deviation of the gaussian distributions from the red cell distribution width (RDW) histograms using the Measure Tool of the ImageJ software (National Institutes of Health, Bethesda, MD, USA). Details on these results are shown in Supplemental Figure S5 and Table 2. Overall, these findings suggest that neutral liposomes do not alter RDW compared to RBCs derived from control mice (Supplemental Figure S5, gray and black lines and bars). However, repeated injections of positively charged LUVs significantly affect RDW (Supplemental Figure S5, blue lines and bars). The data presented in Table 2 reveal a reduction in the average width of RBCs, likely due to the presence of several microcytic RBCs (erythrocytes smaller than usual). As expected, the cationic group displayed the largest standard deviation (Table 2), suggesting a more significant size variability of RBCs, i.e., anisocytosis [27]. This might be explained by the toxic effects exerted by cationic liposomes, which could modify RBCs membrane properties inducing cell adhesions and accelerated removal [28]. However, the alterations in RBCs size might be part of the normal physiological response to the loss of RBCs [29]. Consequently, two events should be occurring in these mice: (i) the faster production of RBCs to compensate low levels results in smaller RBCs due to the high demand for hemoglobin; and (ii) larger RBCs also appear to compensate the loss.

The surface charge has been pointed out as a determining factor affecting the NPs hemocompatibility [30]. Han et al. (2012) [31] revealed that the electrostatic interactions between cationic NPs and the erythrocyte membrane cause erythrocyte agglutination. The functionalization of the NPs surface with negatively charged groups reduced the erythrocyte, aggregating effects of the NPs [31]. In turn, Zhao et al. (2011) revealed that modifying the liposomes surface with PEG molecules may shade the positive charge of the NPs, thus avoiding the toxic blood–liposomes interactions [32].

### 2.8. Organ Weight and Morphology

**Figure 6:**
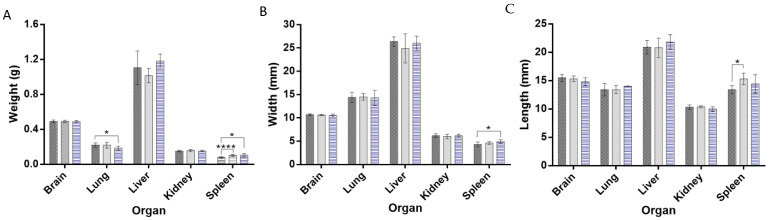
(**A**) Absolute organ weight, (**B**) organ width, and (**C**) organ length of mice treated with repeated doses of PBS (dark gray), neutral (light gray), and cationic (blue) liposomes. * *p* < 0.05, **** *p* < 0.0001.

The comparison of the organ weight between substance-treated and control groups is extensively used to assess harmful effects [33]. In fact, organ weight is one of the most sensitive markers of toxicity since organ damage does not always translate into modifications in its morphology [34]. The absolute weight of the brain, lung, liver, kidney, and spleen was recorded at necropsy (Figure 6A). The brain, liver, and kidney weight and size of mice treated with neutral and cationic liposomes did not significantly change compared to the control group (*p* > 0.05). However, a significant reduction of the absolute lung weight was observed in mice treated with cationic LUVs-treated. Interestingly, the absolute weight of the spleen significantly increased in both liposome-treated groups. This variation was accompanied by an increase in the spleen dimensions in both liposomes-treated groups (*p* < 0.05) (Figure 6B). No major macroscopic changes were observed in the brain, lung, liver, kidney, or spleen after the repeated administration of liposomes.

### 2.9. Histopathological Examination

**Figure 7:**
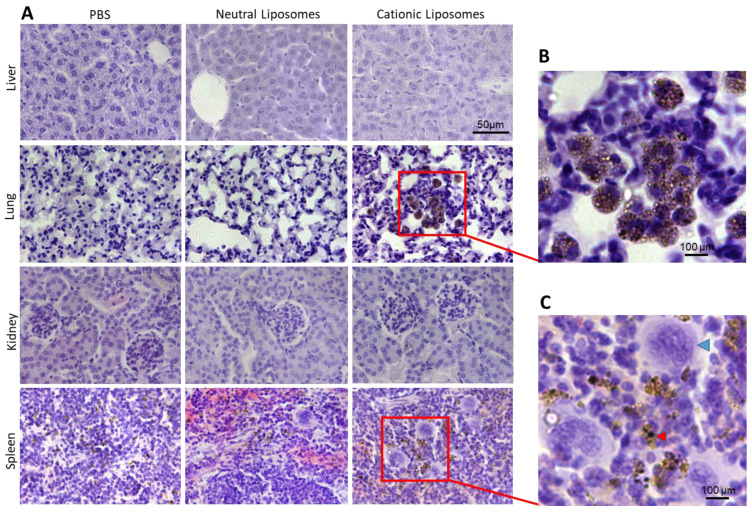
(**A**) Histopathological analysis of liver, lung, kidney, and spleen from mice treated with PBS, neutral, or cationic liposomes (bar depicts 50 µm) (10× magnification). (**B**) Magnification of a lung area from mice treated with cationic liposomes displaying brown pigment (hemosiderin) contained within alveolar macrophages. (**C**) Magnification of a spleen area from mice treated with cationic liposomes depicting brown pigmentation (hemosiderin, red arrowhead) and megakaryocytes (blue arrowhead). Bar in (**B**,**C**) represents 100 µm (40× magnification).

Histopathological studies of several tissues, including liver, lung, kidney, and spleen, were performed. While no alterations were noticed in the tissues of neutral LUVs-treated mice, the histopathological examination revealed that cationic liposomes induce alterations at the hematological level and inflammatory components. The spleen revealed extravascular hematopoiesis observed as diffuse hyperplasia of the red pulp (Supplemental Figure S6). In addition, an evident enrichment in megakaryocytes (Figure 7C, blue arrowhead) was observed in the spleen of mice treated with cationic liposomes compared with animals challenged with neutral liposomes or PBS. While some extra-medullar hematopoiesis is normal in rodents (especially in mice), an increase in this feature may result from induced hematotoxicity. The presence of hemosiderin (brown pigment) (Figure 7C, red arrowhead) in the spleens of cationic liposomes-treated mice may result from hemolytic anemia [35]. It is conceivable that since these are non-PEGylated liposomes, the NPs are recognized relatively fast by the spleen due to binding to proteins such as immunoglobulins, complement proteins and apolipoproteins [18]. Supporting these observations, the lungs of mice treated with cationic liposomes also showed a large population of brown pigmented alveolar macrophages (Figure 7). This could be explained by the high levels of damaged RBCs induced by cationic liposomes. These results agree with the modification of the morphology observed in both organs (Figure 6). Similar findings have been found after the acute administration of cationic nanoparticles [18,36]. Increased DNA damage in both lung and spleen after a single administration of cationic liposomes was observed in a previous study [18]. Moreover, cationic liposomes have been associated with dose-dependent toxicity and pulmonary inflammation. Dokka et al. (2000) demonstrated that cationic liposomes induce the generation of reactive oxygen species in lung cells, causing inflammation and toxicity [7].

Importantly, the above-mentioned alterations are in line with the anisocytosis observed in blood smears (Supplemental Figure S3), the increased hematuria characterized by the presence of shape-altered RBCs in the urine of mice treated with cationic liposomes (Supplemental Figure S2), and the agglutination of fresh blood induced by cationic liposomes in vitro (Figure 4). No evident alterations were observed in the liver and kidneys (Supplemental Figure S6 and Figure 7).

### 2.10. Serum Biochemistry

**Figure 8:**
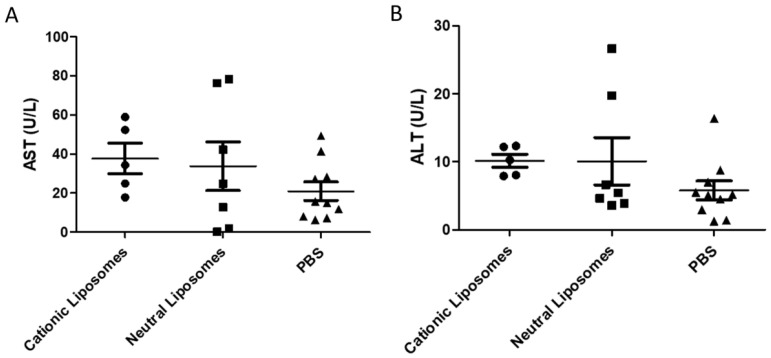
Plasma levels of (**A**) AST and (**B**) ALT in the blood of mice treated with either PBS (triangles), neutral (squares), or cationic liposomes (circles).

The liver is the organ that processes most of the substances introduced into the organism. As such, toxic compounds or molecules may exert their toxicity at the liver level. To explore potential liver-induced toxicity, alterations in the levels of liver proteins such as aspartate aminotransferase (AST) and alanine aminotransferase (ALT) were assessed in the blood of experimental and control mice. In agreement with the data presented in Figure 7, no changes in AST and ALT levels were found between any of the groups included in this study (Figure 8). These data suggest that the liver is not critically damaged by liposomes and support the idea that the toxicity induced by cationic liposomes is mainly associated with RBCs.

## 3. Materials and Methods

### 3.1. Chemicals

1,2-distearoyl-sn-glycero-3-phosphocholine (DSPC, MW 790, CN 850365P), cholesterol (CHOL, MW 387, CN 700000P), 2-distearoyl-sn-glycero-3-phosphoethanolamine-N-[amino(polyethylene glycol)-2000] (ammonium salt) (DSPE-PEG(2000) amine, MW 2791, CN 880128P) and 1,2-dioleoyl-3-trimethylammonium-propane (chloride salt) (DOTAP, MW 699, CN 890890P) were purchased from Avanti Polar Lipids, Inc. (Alabaster, AL, USA). Chloroform was obtained from Sigma-Aldrich (St. Louis, MO, EUA) (CN 25693). Phosphate buffered saline (PBS, 10×, pH 7.0–7.2, 0.067 M, CN SH30256.01) was acquired from GE Healthcare Life Sciences (HyClone™, Logan, UT, USA). Heparin (sodium salt, 1000 units/mL, CN 25021-400-30) was purchased from Sagent (Schaumburg, IL, USA).

### 3.2. Preparation of Liposomes

Neutral liposomes were produced by the thin-film hydration method by sonication [37]. Briefly, DSPC, CHOL, and DSPE-PEG(2000)amine were dissolved in chloroform at a molar ratio of 52:45:3. Then, the organic solvent was evaporated under a nitrogen stream. The produced film was hydrated with PBS at 37 °C (66 mM). To reduce the size, the suspension was sonicated for 40 min (1-min ON, 1-min OFF, 40% of amplitude) in an ice bath using an ultrasonic processor UP400S (Hielscher, Berlin, Germany).

Cationic liposomes were also produced by the thin-film hydration method followed by extrusion [38]. DOTAP and CHOL (molar ratio 85:15) were dissolved in chloroform, and a thin lipid film was obtained after the evaporation of the organic solvent under a nitrogen stream. The dried lipid film was hydrated with PBS at 37 °C (17 mM) and vortexed for 10 min. To reduce the vesicles’ size, the suspension was extruded eleven times through Nuclepore™ (Maidstone, UK) track-etch polycarbonate membranes with a pore size of 100 nm. 

### 3.3. Experimental Animals, Grouping, and Dosing Regime

Thirty-three adult female wild-type mice (C57Bl6) aged between 3–4 months were obtained from Jackson’s laboratories (Bar Harbor, ME, USA). Only female mice were used in the present study to avoid possible variability of the results induced by sex differences. Five to six mice were housed per polypropylene ventilated cage in a room at 22 °C, humidity (40–60%), and a 12/12 h light/dark cycle. Animals were fed with standard mice pellet feed and water ad libitum in the animal facility of the Center for Laboratory Animal Medicine and Care (CLAMC) at UTHealth (Houston, TX, USA). Mice were housed for 48 h before starting the experiments. To promote animal welfare and reproducible experimental results, the rodents were allowed a period of 72 h to acclimate. The animal protocol was reviewed and approved by the Animal Welfare Committee (AWC) of the University of Texas Health Science Center at Houston (UTHealth) (approval number AWC-19-0061). All the experiments were performed according to the institutional guidelines.

The animals were randomly distributed into three groups receiving either vehicle (PBS), neutral or cationic liposomes (11/group). All groups received 10 doses of 200 µL over 3 weeks (administrations held on Mondays, Wednesdays, and Fridays). The doses and duration of treatment were selected according to previous studies [16,18].

### 3.4. Animal Survival, Clinical Observation, Body Weight, and Glucose Levels

Mice were examined daily for clinical signs of toxicity and mortality. The clinical observation included changes in fur, eyes, mucous membranes, lacrimation, and unusual breathing patterns. The body weight (BW) was recorded as an objective measurement before each administration and necropsy. Glucose levels were measured before the first injection and at euthanasia and monitored weekly.

### 3.5. Behavioral Tests

#### 3.5.1. Rotarod Test

The rotarod test was performed before starting the injections and 24 h after the last administration to detect any defects in motor coordination. Following an accelerated speed test protocol, mice were placed into a rotating rod (Med Associates Inc., Fairfax, VA, USA), and the latency to fall was recorded. Succinctly, mice were placed on the rotarod apparatus, rotating at an increasing speed from 4 to 40 rpm, in a trial of 300 s. The latency to fall of 3 trials was recorded. A rest interval of 120 s between each trial was employed to avoid the animals’ fatigue. Two successive animal rotations clinging to the rotarod were registered as latency to fall measurement. Before the first experiment, mice were helped to stay in the correct position for 30 s at a constant speed of 4 rpm to avoid false positives of animals turning, falling, or jumping.

#### 3.5.2. Forelimb Grip Strength Test

Defects on rodents’ limb motor and neuromuscular function were evaluated using a forelimb grip strength test. This test was performed before starting the injections and 48 h post-treatment, allowing an interval rest of 24 h from the rotarod test to avoid mice fatigue. A grip strength meter grid was manufactured to perform this experiment. Concisely, mice were held by the tail and placed horizontally over the grid until the animal forepaws clings. The animals were smoothly pulled back by the tail, and the maximal grip strength value displayed on the screen was recorded. A constant velocity was applied to ensure the repeatability of the test. This procedure was repeated 9 times, with a rest time of 120 s every 3 measurements. The forelimb grip strength values were recorded as the average of the 9 measurements.

### 3.6. Microscopic Urinalysis

In the morning following the last administration, three animals from each group were randomly chosen, and urine was collected for microscopic urinalysis. The microscopic examination was performed using a DMI6000B microscope (Leica, Buffalo Grove, IL, USA). Briefly, a 10 µL drop of urine was placed on a glass microscope slide and covered with a coverslip. The presence of microscopic elements, such as red and white blood cells, epithelial cells, casts, crystals, bacteria, yeast, and clumps, was analyzed.

### 3.7. In Vitro Interaction between Blood and Liposomes

The blood was collected by cardiac puncture after being euthanized by CO_2_ inhalation. Blood samples (200 µL) were mixed with 200 µL of DSPC:CHOL:PEG(2000) amine and DOTAP:CHOL LUVs (1:1 *v*/*v*). A control sample was prepared containing an identical volume of PBS. Blood turbidity changes, clot formation, and hemolysis were examined macroscopically. Then, blood smears of the mixtures (30 µL) were performed and observed using a DMI6000B microscope (Leica, Buffalo Grove, IL, USA).

### 3.8. Acute In Vivo Interaction between Blood and Liposomes

A group of adult female wild-type mice were injected with a single dose of DOTAP:CHOL LUVs. Animals were euthanized by an overdose of anesthesia, and the blood was collected by cardiac puncture. Animals injected with PBS were subjected to the same procedure and used as controls. Blood smears from both animal groups (30 µL) were performed and observed using a DMI6000B microscope (Leica, Buffalo Grove, IL, USA). The effect of a single dose of positively charged liposomes on the morphology of RBCs was evaluated.

### 3.9. Prolonged In Vivo Study to Assess the Interaction between Blood and Liposomes

After administering 10 doses of PBS, DSPC:CHOL:PEG(2000) amine, and DOTAP:CHOL LUVs, the surviving animals were euthanized 120 h after the last injection via CO_2_ inhalation followed by cardiac puncture to collect the blood. Three blood samples (30 µL) of each group were randomly selected to perform blood smears. The morphology of RBCs was analyzed using a DMI6000B microscope (Leica, Buffalo Grove, IL, USA).

### 3.10. Necropsy, Organ Weight, and Morphology

Necropsy was performed immediately after euthanasia. Here, each animal’s brain, lungs, liver, kidneys, and spleen were excised and examined macroscopically to identify possible signs of toxicity. The organs were washed with PBS, placed on an absorbent paper for a few seconds, and the absolute organ weight was determined. The dimensions of the organs were also recorded using an automatic digital caliper (Neiko, Wenzhou, China). For further histopathological studies, the organs were placed in a 10% neutral buffered formalin solution. The blood samples from cardiac punctures were collected into Eppendorf tubes containing sodium heparin as an anticoagulant. Blood samples were centrifuged at 3000 rpm for 15 min to collect the serum. Plasma samples were frozen in liquid nitrogen and then stored at −80 °C until used for the liver injury assessment. 

### 3.11. Histopathological Examinations

Major organs were stored in 3.7% formaldehyde, paraffin-embedded, and sliced at 10 μm for hematoxylin-eosin staining following routine protocols [39]. Briefly, after deparaffinization and rehydration, tissue slices were stained with hematoxylin solution for 5 min in a dark container and rinsed with distilled water. Next, sections were stained with eosin solution for 30 s, followed by dehydration with alcohol and clearing with xylene. The mounted sections were visualized under light microscopy, and histopathological evaluation was performed for spleen, lung, liver, and kidney tissues. Stained slices were observed at low- and high-power magnifications (10× and 40×, respectively) using a Leica DMI6000 B microscope (Leica Microsystems, Buffalo Grove, IL). Representative photomicrographs were taken with a digital camera (DFC310FX Leica™, Wetzlar, Germany). Low- and high-power magnifications were used to qualitatively analyze the cell type content in the red pulp of the spleen, the micromorphological integrity, the presence of granuloma formation, and the extent of cellular infiltration exhibited by lungs, livers, and kidneys.

### 3.12. Quantification of Liver Injury Markers in Blood

The levels of two established liver injury biomarkers, ALT and AST, were measured via diagnostic enzyme assay kits (ATL kit: catalog # A524-150, lot 86154; AST kit: catalog # A559-150, lot 84022, both Teco Diagnostics, Anaheim, CA, USA) in blood plasma, according to the manufacturer’s protocol. ALT and AST activity was determined by measuring the rate of oxidation of NADH throughout an enzymatic reaction sequence at a specific wavelength (340 nm) using a SpectraMax^®^ iD3 Multi-Mode Microplate Reader (Molecular Devices, San José, CA, USA).

### 3.13. Statistical Analysis

All the results are expressed as mean ± standard deviation. The statistical analysis of data was performed using student’s t-tests, with a confidence interval of 95%. Results whose *p*-values ≤ 0.05 were considered significantly different. Statistical analysis and data presentation were completed using Prism 8 (GraphPad Software Inc. La Jolla, CA, USA).

## 4. Conclusions

This study thoroughly characterized the potential toxicity of neutral and cationic liposomes after repeated intravenous administrations in rodents. Our results show that cationic liposomes are toxic, evidenced by a 45% lethality. According to the results, the toxicity induced by the repeated administration of the cationic liposomes appears to be associated with the adverse interactions of the lipid vesicles with anionic serum macromolecules. Consequently, substantial changes in the spleen and liver of the mice were noticed. Therefore, caution should be exercised when using cationic liposomes in vivo. Importantly, our results highlight the safety of neutral liposomes and support their use as drug shuttles, even when repeated doses of a therapeutic agent are needed to be administered for optimal effects.

## Figures and Tables

**Table 1:** Physicochemical properties of neutral and cationic liposomes in terms of hydrodynamic diameter, polydispersity index (PDI), and zeta potential.

Liposomes	Composition	Hydrodynamic Diameter (nm)	PDI	Zeta Potential (mV)
Neutral	DSPC:CHOL:DSPE-PEG(2000) amine	139 ± 13	0.22 ± 0.02	−1 ± 1
Cationic	DOTAP:CHOL	112 ± 3	0.13 ± 0.01	33 ± 2

**Table 2:** Effect of the repeated administration of PBS, neutral, and cationic liposomes on the mean width of red blood cells (RBCs).

	PBS Group	Neutral Group	Cationic Group
Mean width of RBCs (pixel)	44.7 ± 2.6	44.6 ± 2.6	43.4 ± 3.1

## Data Availability

Data is contained within the article and Supplementary Material.

## Supplementary Materials

The following supporting information can be downloaded at: https://www.mdpi.com/article/10.3390/ph15060761/s1, Figure S1: Effect of the repeated administration of PBS (dark gray), neutral (light gray), and cationic liposomes (blue) on blood glucose levels; Figure S2: Microscopic urinalysis of mice treated with cationic and neutral liposomes; Figure S3: Microscopic images of blood smears after mixing blood and liposomes ex vivo; Figure S4: Acute in vivo cationic liposomes-blood interaction; Figure S5: Effect of prolonged administration of PBS (black), neutral (gray), and cationic liposomes (blue) on the red blood cell distribution width (RDW); Figure S6: Histopathological analysis of liver, lung, kidney, and spleen at low power magnification; Table S1: Effect of the repeated administration of PBS, neutral and cationic liposomes on mice body weight.
