# Supplementary material for: Multi-Dose Intravenous Administration of Neutral and Cationic Liposomes in Mice: An Extensive Toxicity Study
Source: Pharmaceuticals (Basel). 2022 Jun 18;15(6):761. doi: 10.3390/ph15060761 (PMC9229811; doi:10.3390/ph15060761)
Supplement: Supplementary file 1 [file pharmaceuticals-15-00761-s001.zip › pharmaceuticals-1751234-supplementary.pdf]

SUPPLEMENTAL INFORMATION

Multi-dose intravenous administration of neutral and cationic liposomes in mice: an extensive toxicity study

Stéphanie Andrade*, Joana A. Loureiro*, Santiago Ramirez, Celso S. G. Catumbela, Claudio Soto, Rodrigo Morales, Maria Carmo Pereira

* These authors contributed equally to this work.

Supplemental Table S1. Effect of the repeated administration of PBS, neutral and cationic liposomes on mice body weight.

	Body weight (g)		
**Injection**	**PBS group**	**Neutral group**	**Cationic group**
1	21.6 ± 1.3	21.6 ± 1.4	21.2 ± 0.8
2	21.9 ± 1.4	20.4 ± 1.3	21.4 ± 0.4
3	21.6 ± 1.5	21.5 ± 1.1	21.0 ± 0.6
4	21.5 ± 1.4	21.3 ± 1.1	21.0 ± 0.6
5	21.6 ± 1.4	21.0 ± 1.0	21.4 ± 0.6
6	21.3 ± 1.4	21.0 ± 1.1	20.9 ± 0.6
7	21.5 ± 1.5	21.0 ± 1.1	21.1 ± 0.4
8	21.9 ± 1.7	21.6 ± 1.1	21.8 ± 0.6
9	21.8 ± 1.4	21.5 ± 1.1	21.8 ± 0.7
10	21.8 ± 1.9	21.3 ± 1.0	21.7 ± 0.5
End	21.9 ± 1.7	21.5 ± 1.0	21.5 ± 0.8

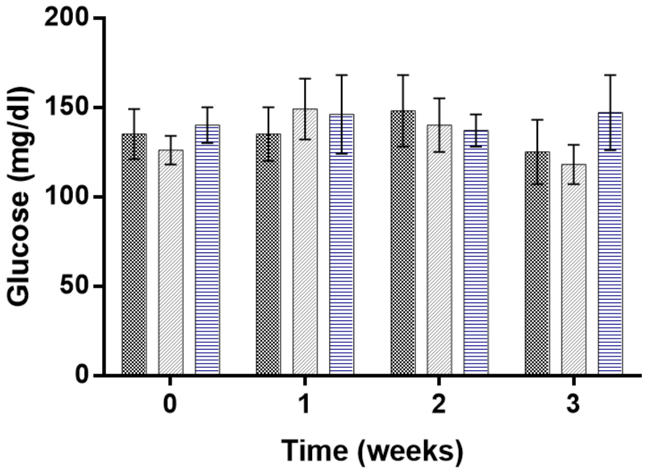

Supplemental Figure S1. Effect of the repeated administration of PBS (dark gray), neutral (light gray), and cationic liposomes (blue) on blood glucose levels.

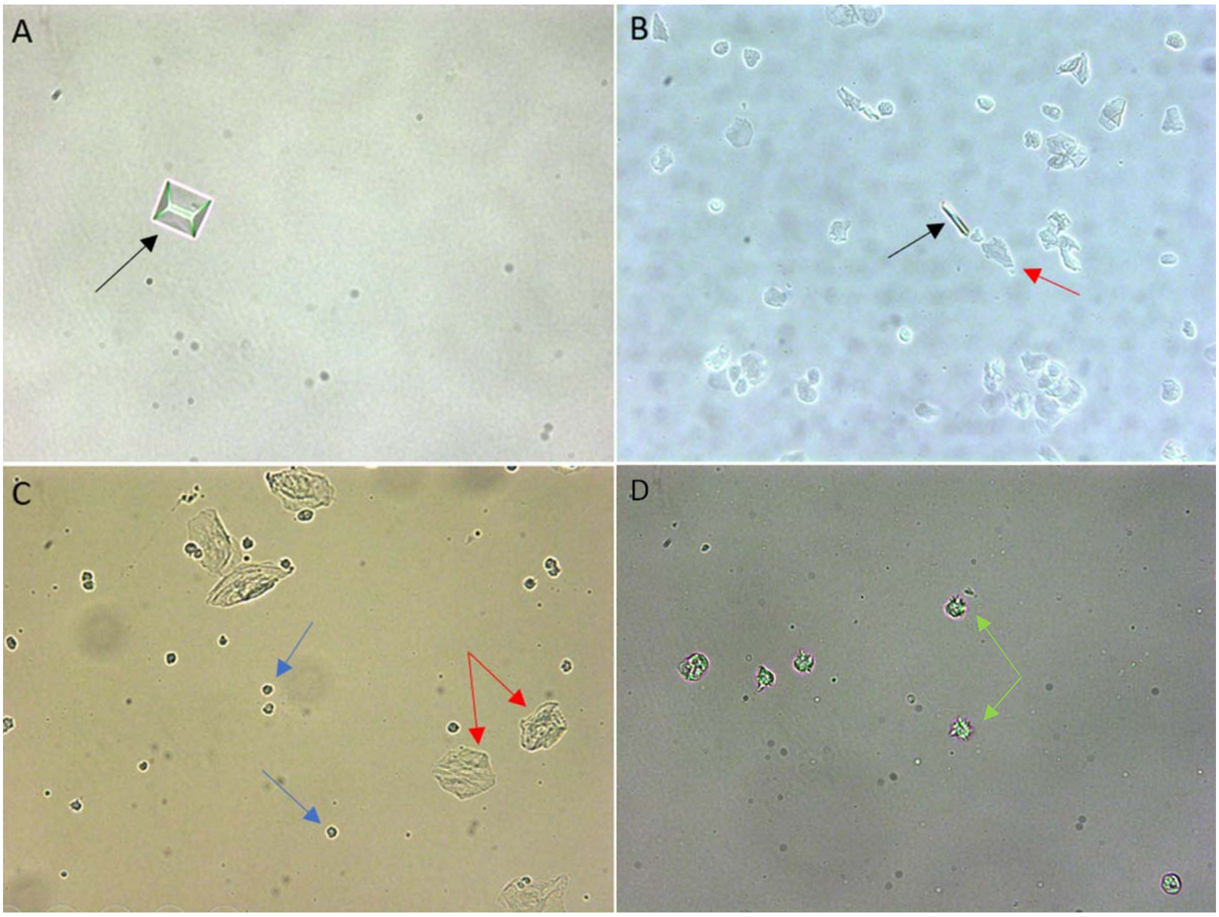

Supplemental Figure S2. Microscopic urinalysis of mice treated with cationic and neutral liposomes. Urine from mice subjected to repeated doses of PBS (A), neutral liposomes (B), and cationic liposomes (C and D) were evaluated under the microscope. Magnifications used in each panel are A) 40X, B) 10X, C) 20X, D) 40X. Black arrows: triple phosphate crystals; blue arrows: RBCs; red arrows: endothelial cells.

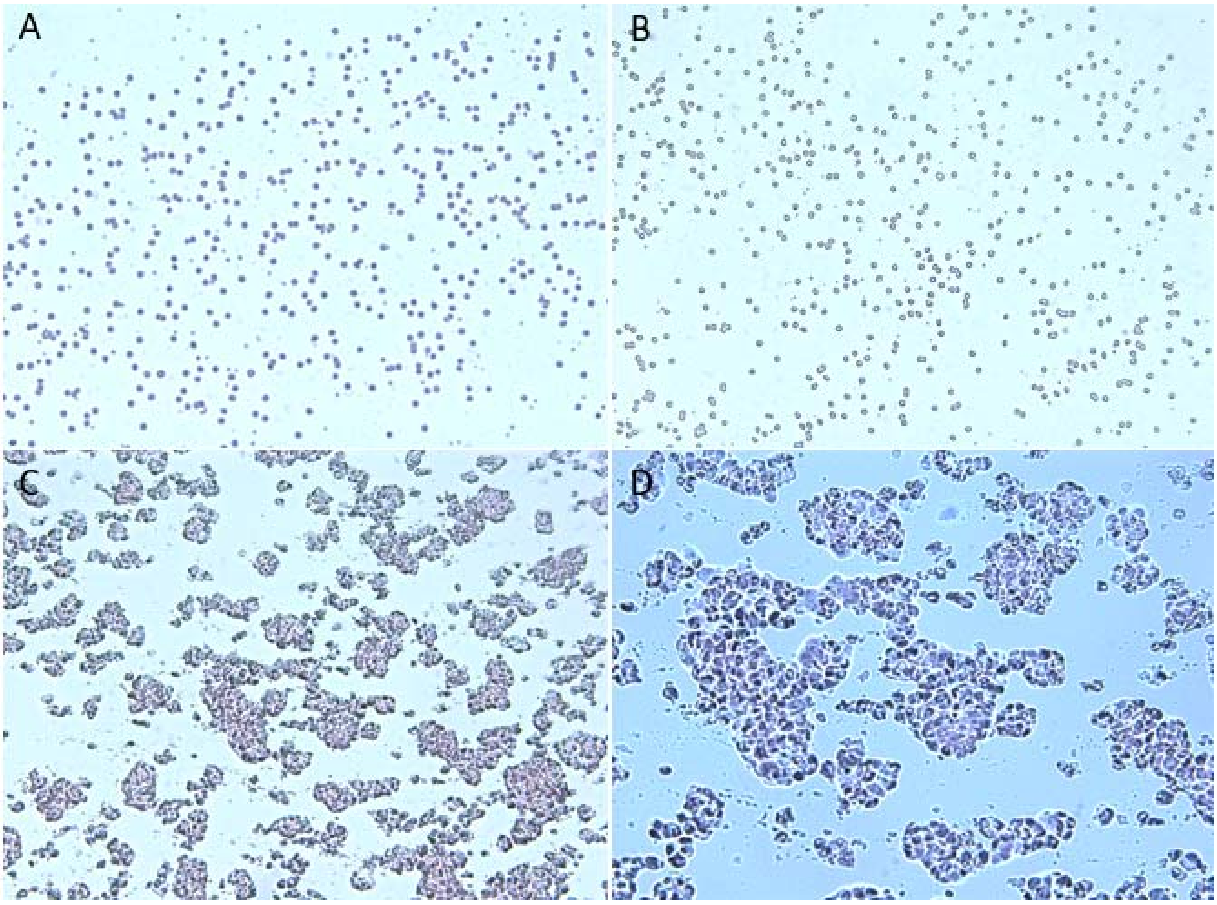

Supplemental Figure S3. Microscopic images of blood smears after mixing blood and liposomes *ex vivo*. Blood samples from the experiment described in Figure 7 were checked under the microscope. Panels represent mouse blood mixed with PBS (A), neutral liposomes (B), and cationic liposomes (C and D). Magnifications used in each panel are as follows: A) 10X, B) 10X, C) 10X, D) 20X.

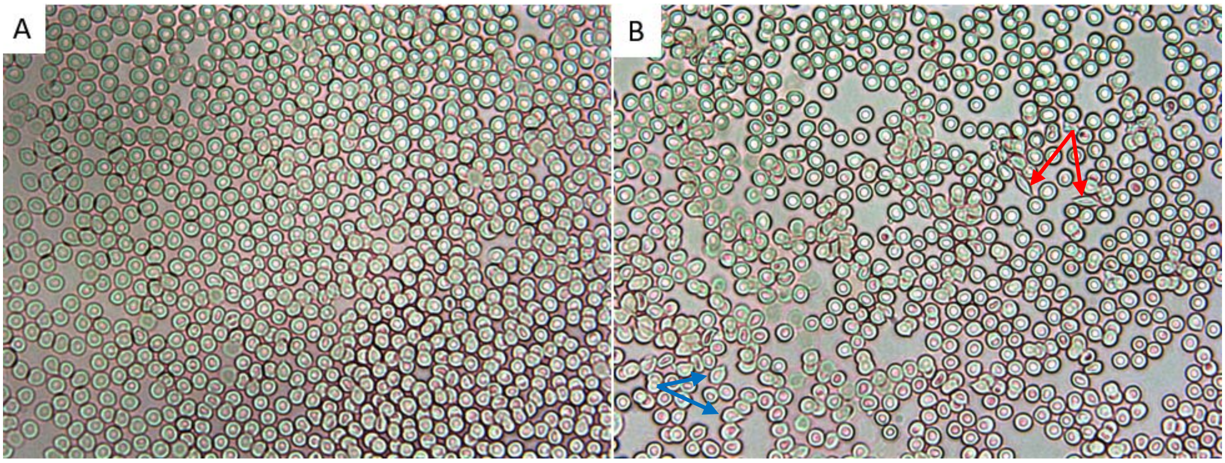

Supplemental Figure S4. Acute *in vivo* cationic liposomes-blood interaction. Microscopic images of blood smears before (A) and after (B) injecting one dose of DOTAP:CHOL LUVs into mice. Images in this figure were obtained at a 40X magnification. Blue arrows represent dacrocytes, and red arrows depict acuminocytes.

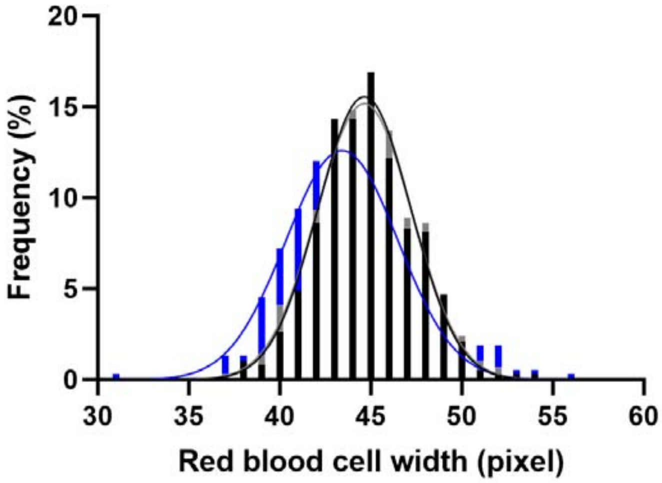

Supplemental Figure S5. Effect of prolonged administration of PBS (black), neutral (gray), and cationic liposomes (blue) on the red blood cell distribution width (RDW). Data corresponds to the frequency distribution of RBCs width measured in pixels in a blood smear as an indicator of anisocytosis.

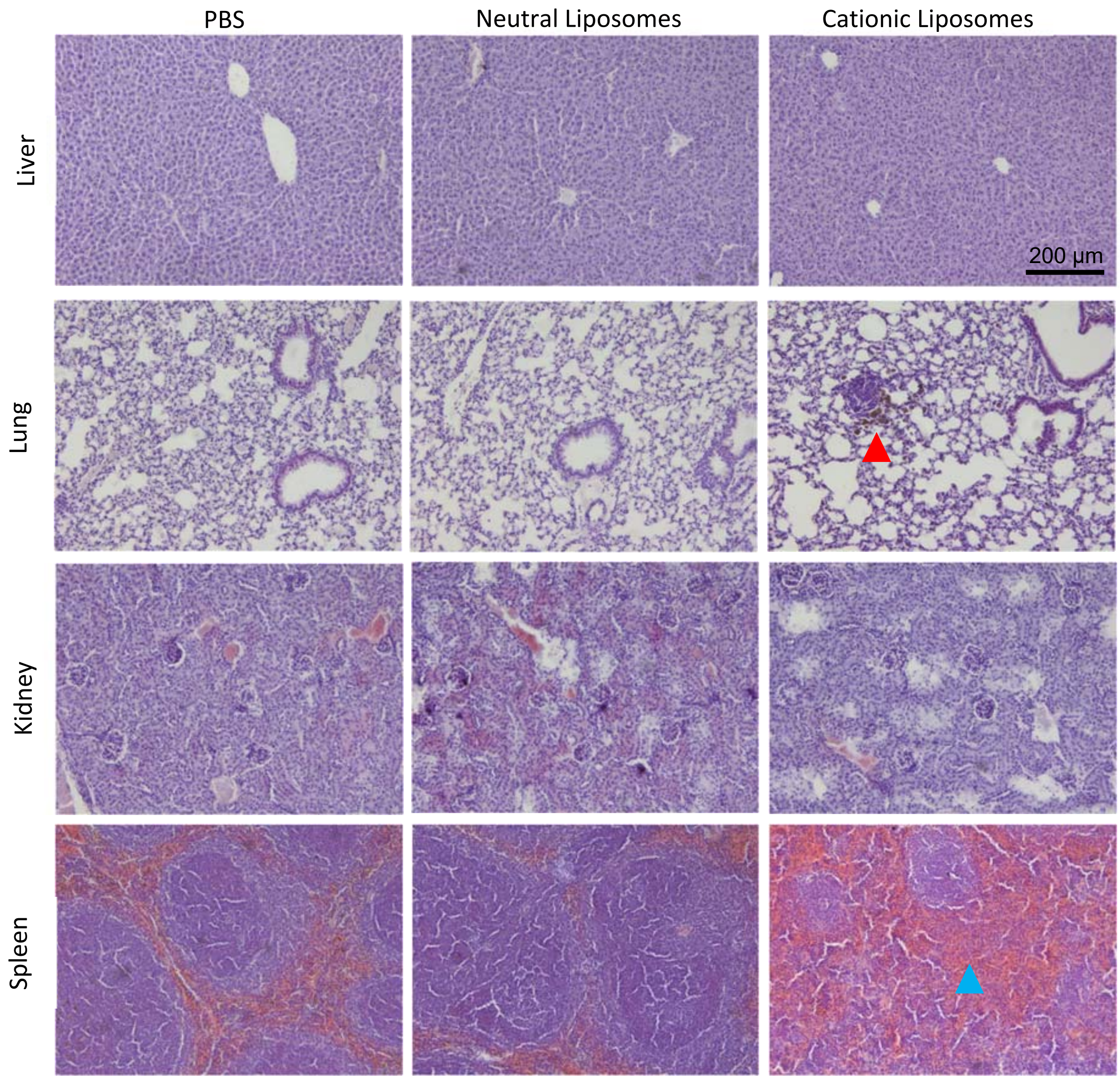

Supplemental Figure S6. Histopathological analysis of liver, lung, kidney, and spleen at low power magnification. A comparison between different experimental groups after treatment shows the presence of brown pigmented cells only in animals treated with cationic liposomes (red arrowhead). In spleens of mice treated with cationic liposomes, an increased area of the red pulp (blue arrowhead) was observed. Images in this figure were obtained at a 10X magnification**.**
